# Erythrocytes: Central Actors in Multiple Scenes of Atherosclerosis

**DOI:** 10.3390/ijms22115843

**Published:** 2021-05-29

**Authors:** Chloé Turpin, Aurélie Catan, Olivier Meilhac, Emmanuel Bourdon, François Canonne-Hergaux, Philippe Rondeau

**Affiliations:** 1 Diabète Athérothrombose Thérapies Réunion Océan Indien (DéTROI), INSERM, UMR 1188, Université de La Réunion, 97400 Saint Denis, France; chloe.turpin@univ-reunion.fr (C.T.); aureliecatan5586@gmail.com (A.C.); olivier.meilhac@inserm.fr (O.M.); emmanuel.bourdon@univ-reunion.fr (E.B.); 2 Centre Hospitalier Universitaire de La Réunion, 97400 Saint Denis, France; 3 IRSD, Université de Toulouse, INSERM UMR1220, INRA UMR1416, ENVT, UPS, 31000 Toulouse, France; francois.canonne-hergaux@inserm.fr

**Keywords:** atherosclerosis, erythrocytes, erythrophagocytosis, eryptosis, oxidative stress, glycation, iron, heme, haemoglobin

## Abstract

The development and progression of atherosclerosis (ATH) involves lipid accumulation, oxidative stress and both vascular and blood cell dysfunction. Erythrocytes, the main circulating cells in the body, exert determinant roles in the gas transport between tissues. Erythrocytes have long been considered as simple bystanders in cardiovascular diseases, including ATH. This review highlights recent knowledge concerning the role of erythrocytes being more than just passive gas carriers, as potent contributors to atherosclerotic plaque progression. Erythrocyte physiology and ATH pathology is first described. Then, a specific chapter delineates the numerous links between erythrocytes and atherogenesis. In particular, we discuss the impact of extravasated erythrocytes in plaque iron homeostasis with potential pathological consequences. Hyperglycaemia is recognised as a significant aggravating contributor to the development of ATH. Then, a special focus is made on glycoxidative modifications of erythrocytes and their role in ATH. This chapter includes recent data proposing glycoxidised erythrocytes as putative contributors to enhanced atherothrombosis in diabetic patients.

## 1. Introduction

Erythrocytes appear as basic simple anucleated cell tramps travelling in the blood, with a role limited to oxygen (O_2_) and carbon dioxide (CO_2_) carriage between organs and lungs. Indeed, gas transportation represents the main physiological role attributed to erythrocytes. In ischaemic vascular disease, erythrocytes are often reduced to their participation in the clot, obstructing blood vessels.

Atherothrombosis is the leading cause of morbidity and mortality in the developed world and represents the ultimate dramatic evolution of atherogenesis, which is characterised by the formation of a thrombus and eventually vessel occlusion [1,2].

**Figure 1:**
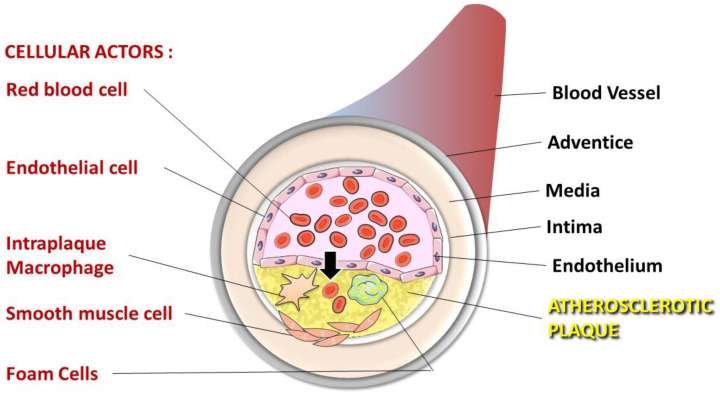
Cellular actors present in the atheroma plaque. Dysfunction of endothelial cells allows lipoprotein and erythrocyte infiltration in the sub-endothelial space (the arrow indicates this infiltration). Intraplaque macrophages that engulf oxidised LDL and infiltrated erythrocytes differentiate into foam cells initiating the atheroma formation. Enhanced inflammatory processes in atheroma cause smooth muscle cell migration from the media to the intima, towards the necrotic core formed by dead foam cells.

The formation of atherosclerosis (ATH) has clearly been shown to involve lipid accumulation and oxidative stress phenomena. Lipoprotein infiltration, oxidation and accumulation within the vessel intima are well-documented phenomena contributing to atherogenesis. In the progression of this vascular pathology, erythrocytes should not be considered as simple bystanders. In addition to the pivotal role that erythrocytes may play in the onset, progression and complication of ATH, the role of their released components and subsequent intravascular haemolysis, as well as the increased interaction of erythrocytes with vascular cells, is not fully understood. Along with other cells present in the atheroma plaque (Figure 1), erythrocytes were recently described as putative key players in the formation and complications of atheromas [3,4].

Erythrocyte phagocytosis by cells present in the plaque of the atheroma can represent an important source of local homeostasis imbalance in terms of lipids, iron and oxidative damage. This review focuses on the role of erythrocytes in atherogenesis and atherothrombotic complications. Erythrocyte physiology (Section 2) and the natural history of ATH (Section 3) are first described. Section 4 delineates the numerous links between erythrocytes and atherogenesis. In particular, the impact of extravasated erythrocytes on plaque iron homeostasis with pathological consequences is addressed. In addition, hyperglycaemia is now well recognised as a major contributor to the development of ATH. In fact, in hyperglycaemic conditions, such as those encountered in diabetic patients, erythrocytes are subjected to glycoxidative modifications, which render them more prone to interacting with vascular cells. Therefore, we include a subchapter on recent data focusing on glycated erythrocytes and their link to enhanced atherothrombosis in diabetic patients.

## 2. Erythrocytes—Simple, Vital and Fragile Cells

### 2.1. Main Features and Functions

**Figure 2:**
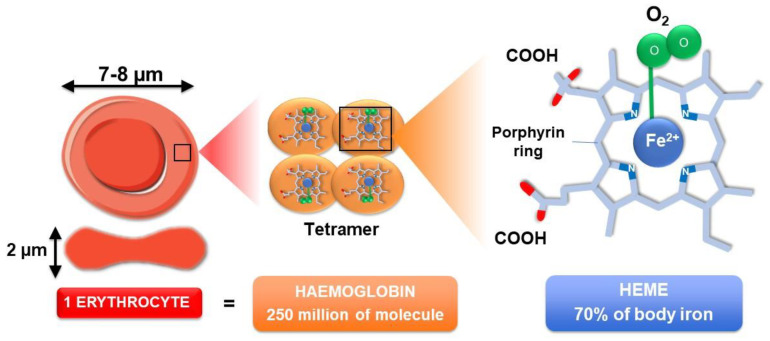
Erythrocyte structure and content. One single erythrocyte contains about 250 million haemoglobin molecules. Haemoglobin, the protein responsible for oxygen transport, is a tetramer composed of 4 globin chains. Each globin chain contains one heme molecule responsible for the binding of one oxygen molecule (O_2_). Heme consists of a porphyrin ring centred by one atom of iron (Fe^2+^). Oxygen strongly binds to this iron atom. Almost two-thirds of the body iron (about 2.5 to 36 g) is localised in haemoglobin of circulating erythrocytes.

Erythrocytes represent the most abundant cell type in the human body with about 5 × 10^6^ cells/mm^3^ of blood (for a healthy person). They are continuously produced (2.4 × 10^6^ cells/second) in the bone marrow. After maturation, erythrocytes live up to 120 days, and then senescent cells are removed from the circulation by myeloid phagocytic cells (macrophages) present in the spleen, liver and bone marrow [5]. Erythrocytes consist of concentrated haemoglobin enveloped in a flexible membrane and display unique structural features, without a nucleus, mitochondria or ribosomes [6]. Their typical biconcave disc shape, with an average diameter of 7 to 8 µm and a thickness of 2 µm, renders erythrocytes perfectly suited for gas transportation and exchange (CO_2_ and O_2_) between lungs and tissues [7] (Figure 2). The vital function of O_2_ and CO_2_ transportation in the blood is endorsed by haemoglobin, representing more than 95% of erythrocyte cytoplasmic proteins [8]. With about 250 million molecules per erythrocyte, haemoglobin represents the most widespread heme-containing proteins in the body and displays an allosteric tetrameric structure consisting of two alpha chains and two β chains.

Each of the four globins is attached to a heme group containing one iron atom that can bind to one O_2_ molecule. With a concentration of about 3.5 mg of iron per g of haemoglobin (Hb), erythrocyte constitutes the primary source of iron in the body (around 70% which correspond to 2.5 to 3 g of iron) (Figure 2). The reduced ferrous form (Fe^2+^) contained in haemoglobin (oxyHb or ferroHb) allows O_2_ transport and distribution to all tissues.

In physiological conditions, about 3% of haemoglobin can be oxidised into metheamoglobin (metHb or ferriHb), an altered form of haemoglobin containing a heme iron in the ferric state (Fe^3+^) [9]. When metheamoglobinaemia occurs (amount of metheamoglobin above 3%), haemoglobin is not able to effectively release the O_2_ that it carries.

The oxygenation status of erythrocytes determines the conformational changes in haemoglobin molecules and consequently allows O_2_ and CO_2_ binding. Due to its composition and function in the circulation, erythrocytes participate in the regulation of the redox homeostasis of its direct environment. Despite the absence of mitochondria in erythrocytes, many reactive oxygen species (ROS) are continuously produced, such as the superoxide anion (O_2_^•−^), hydrogen peroxide (H_2_O_2_) and hydroxyl radical (HO^•^) [10]. The intracellular production of ROS could be caused by the continuous autoxidation of the O_2_ carrier haemoglobin due to the high O_2_ tension and from the iron ion associated with the protein moiety [10,11,12]. Autoxidation of this ferrous ion present in oxygenated haemoglobin (ferroHb also referred to as HbO_2_) induces the release of O_2_^•−^ and the formation of ferric haemoglobin (ferriHb), which could be subsequently oxidised by strong oxidants, such as H_2_O_2,_ into a highly reactive intermediate ferryl haemoglobin (ferrylHb (iron in a ferryl state (Fe^4+^)) [13]. Under certain pathological conditions (e.g., sickle cell disease) and also in physiological conditions, endogenous ROS production could also be mediated enzymatically by the pro-oxidant enzyme NADPH oxidase [14,15]. This enzyme is the primary source of O_2_^•−^ production in many cells. In parallel with those produced within the cytosol of erythrocytes, ROS could be encountered in the circulation [16]. The membrane of the erythrocyte is effectively permeable to superoxide anion and hydrogen peroxide via the membrane transport protein band.3 [17,18]. As a result, erythrocytes could be considered as free radical scavengers in the plasma [16].

### 2.2. Erythrocyte Membranes Exhibit Specific Features

The vital necessity of gas supply to organs requires the passage of erythrocytes through tiny capillaries, whose diameter is half of their own. Thus, erythrocyte passage without disruption implies great elasticity and elongation of membrane capacity [19]. Erythrocyte deformability totally depends on the structure of the cell membrane. The membrane of the erythrocyte is composed of a phospholipid bilayer with a large amount of associated and encased proteins. Proteins represent half the amount of biological constituents of the membranes of erythrocytes. Carbohydrates (about 8% of the membranes of erythrocytes) make glycosyl adducts on lipids and proteins of the membrane of the erythrocyte. Lipids in the membranes of erythrocytes are mainly phospholipids and unesterified cholesterol [20].

As in all eukaryotic membranes, the lipid bilayer includes various types of phospholipids, which are asymmetrically distributed across the bilayer. Sphingolipids and choline phospholipids are mainly concentrated on the outer layer, while amino phospholipids (phosphatidylserine and phosphatidylethanolamine) are localised on the inner leaflet of the bilayer [20]. The coherence and stability of the bilayer are possible through the asymmetric distribution of these constituent phospholipids. The maintenance of the distribution and asymmetry of lipids is ensured through interactions involving aminophospholipids in the inner leaflet of the bilayer and the cytoskeleton-associated proteins [21,22]. Changes in the composition or distribution of lipids in the membranes of erythrocytes result in morphological abnormalities and/or impairment in the capacity for membrane deformability that can lead to a reduced cell life span or to an altered blood rheological pattern [23,24,25]. Hence, this asymmetric distribution is of great importance for the structural and functional properties of erythrocytes.

**Figure 3:**
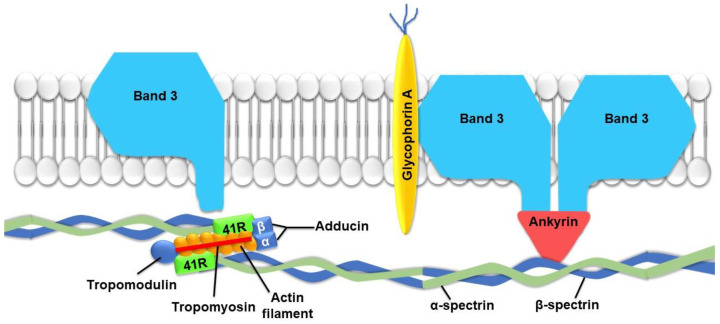
Schematic representation of important proteins involved in erythrocyte membrane flexibility. Spectrin molecules, responsible for stability and elasticity, are attached to the cell membrane by ankyrin. 41R protein establishes a link between actin and spectrin filaments.

The skeleton of the erythrocyte exhibits specific characteristics, such as the absence of the structural protein tubulin. The role of the skeleton of the erythrocyte is mainly devoted to the shape of the cell and its capacity for deformability [26]. Specific proteins in the cytoskeleton of the erythrocyte, such as spectrin, protein 4.1, F-actin and ankyrin, play determinant roles in the regulation of cell deformability. Indeed, the four proteins are closely associated with the creation of a filamentous network, connecting proteins present in the membrane and ensuring cellular shape and elasticity [27]. The spectrin–actin–protein 4.1 complex constitutes the morphological properties of the cell, while ankyrin provides an anchorage to the bilayer via membrane proteins, such as the anion exchange protein band.3 [28,29] or glycophorins [30] (Figure 3). In addition to its significant involvement in the elasticity and stability of the membranes of erythrocytes, band.3 protein also plays a key role in mediating O_2_-regulated metabolic transitions [31].

The essential deformability capacity of erythrocytes may be influenced by microenvironmental (pH and osmolarity) and pathophysiological conditions (chronic hyperglycaemia and inflammation). Indeed, reduced erythrocyte deformability, in relation to microvascular diseases, has been reported in several studies [32,33,34]. Impairments of erythrocyte deformability may effectively cause significant disturbances in micro- and macrocirculation, producing an increase in flow resistance and blood viscosity [35]. In particular, there is consistent evidence that diabetes and other pathological conditions, such as sickle cell disease, induce alterations in the structure and function of erythrocytes through an alteration in deformability [33,36,37]. This loss of the membrane fluidity and deformability of the erythrocyte was associated with enhanced cell fragility and susceptibility to haemolysis when cells were placed in a high-shear stress environment [38].

### 2.3. Erythrocytes Are Subject to Haemolysis

During their 120-day life span, erythrocytes have to pass through tiny capillaries many times. While transporting O_2_ to tissues, erythrocytes are highly exposed to oxidative stress-mediated damages, challenging both the structure and function of the erythrocyte and reducing its lifespan. During ageing, erythrocytes develop an enhanced susceptibility to haemolysis. Erythrocyte ageing or senescence or eryptosis is notably marked by changes in the activity of a number of intracellular enzymes due to the lack of renewal. An impairment of the activity of certain enzymes ((Ca^2+^, Mg^2+^)-ATPase) could induce an intracellular increase in Ca^2+^ and a decrease in potassium chloride (KCl). This modification in intracellular ion balance may lead to enhanced cell dehydration, with a consequent loss of the membrane’s capacity to protect haemoglobin from irreversible oxidation [39].

**Figure 4:**
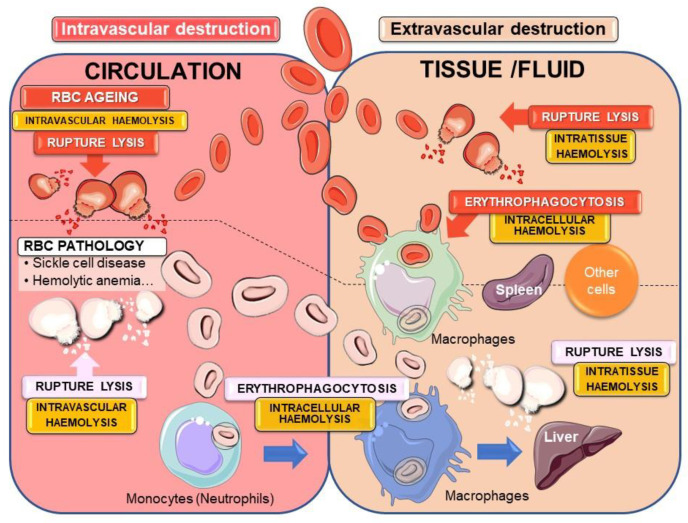
Physiological and pathological intra- and extravascular destruction of erythrocytes. The fate of senescent (or eryptotic) red blood cells (RBCs) can undergo different pathways for cellular destruction. Rupture lysis into the blood circulation (intravascular haemolysis) or into tissues and fluids (intratissue haemolysis) in the case of RBC intravasation can occur with the liberation of hemolytic by-products. To avoid such release of harmful components, the major mechanism of senescent erythrocyte removal passes through the process of erythrophagocytosis by macrophages (intracellular haemolysis) mainly in the spleen. In many erythrocyte pathologies, the process of eryptosis is accelerated leading to massive elimination of erythrocytes both by rupture lysis and erythrophagocytosis into the circulation or inside tissues/fluid as well. Pathological erythrocytes can be recognised and phagocytosed by circulating monocytes that could migrate to tissues such as the liver.

The term haemolysis is mainly (or only) defined as the rupture of the erythrocyte’s membrane with the release of haemolytic by-products, such as haemoglobin, heme, iron or ROS. Accordingly, intravascular haemolysis refers essentially to rupture lysis in the blood with the release of haemolytic by-products. On the other hand, extravascular haemolysis is described in the ingestion (phagocytosis) and destruction of the erythrocyte in macrophages of the spleen, an erythrophagocytosis (EP) process that prevents the release of harmful erythrocyte components. The term “extravascular haemolysis” could be misleading and could cause confusion to the readers according to the definition of haemolysis in medical textbooks. In addition, the global picture of erythrocyte destruction (a term more appropriate in this context) is more complex and needs some clarification and likely a new definition, as presented in Figure 4. Haemolysis could be defined as the lysis of erythrocytes either by rupture or erythrophagocytosis, with precision of its specific localization (intravascular, intratissue/fluid or intracellular) (Figure 4).

After the physiological process of ageing (eryptosis see Section 2.3.2), erythrocytes can undergo either intravascular or extravascular destruction. The former corresponds to rupture lysis into the circulation and can be defined as intravascular haemolysis. Extravascular destruction could occur with the entry of erythrocytes into tissues or fluids (i.e., tumours, brain with haemorrhagic stroke, or haemorrhagic atheromatous plaque). Such extravascular destruction of extravasated erythrocytes happens by rupture lysis that can be defined as intratissue haemolysis. However, due to specific changes in erythrocytes during eryptosis, the main destruction of erythrocytes is achieved through the process of EP (engulfment and intracellular degradation), mainly by splenic macrophages, but other cells present in the haemorrhagic tissues could also contribute to the process. Indeed, eryptosis could be considered as a useful and safety mechanism for the removal of defective erythrocytes to prevent release. The destruction of erythrocytes by splenic macrophages has been often defined as extravascular haemolysis, a very ambiguous definition, since it does not take into account intratissue haemolysis. Therefore, we proposed this process to be defined as intracellular haemolysis.

In pathological situations affecting the life of red blood cells (RBCs) with acceleration of the eryptosis process, such as in sickle cell disease, haemolytic anaemia due to erythrocyte enzyme deficiencies (such as G6PD or pyruvate kinase), or in diabetes with erythrocyte glycation, erythrocytes are fragile and more prone to both intra- and extravascular destruction. In addition, in such conditions, EP can occur in monocytes (or neutrophils) present in the circulation, with subsequent migration into the liver [40].

#### 2.3.1. Intra- or Extravascular Haemolysis with Erythrocyte Rupture Lysis

When intra- or extravascular haemolysis occurs, erythrocytes lose their integrity and release their contents, including haemoglobin degradative products (such as heme and iron), into the peripheral circulation or into the tissues, respectively.

**Figure 5:**
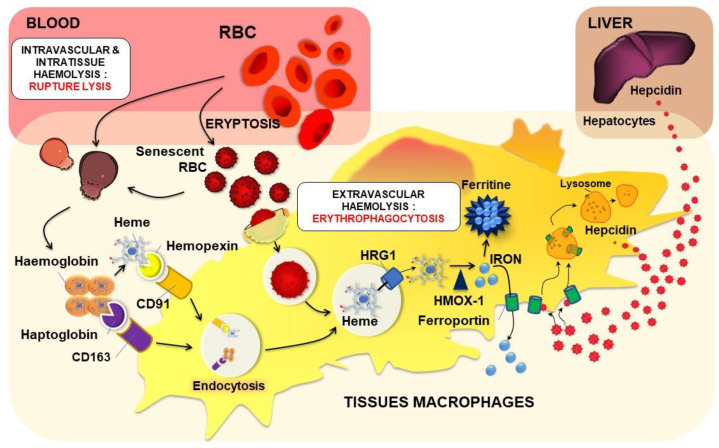
Erythrocyte haemolysis and heme iron recycling. Modifications (oxidative stress, glycation; see chapter IV) and/or eryptosis can trigger the lysis of circulating or extravasated erythrocytes in some tissues. After such intravascular or intratissue haemolysis, haptoglobin and hemopexin bind to haemoglobin and heme, respectively. Complexes are recognised by macrophages and endocytosed via their specific receptors CD163 for haemoglobin/haptoglobin complexes and CD91 for heme/hemopexin complexes. In the case of EP of senescent or eryptotic erythrocytes (intracellular haemolysis), specific recognition of old erythrocytes is performed by tissue macrophages, and a phagolysosome containing the old erythrocyte is formed. Both CD163- and CD91-mediated endocytosis and EP lead to the formation of a vacuole containing heme. Heme is then transported to the cytosol via HRG1 and degraded by HMOX1 (Heme oxygenase 1) to produce iron. According to the body needs, such iron is either stored in ferritin or transported outside the cells via the transporter ferroportin. Hepcidin, a small but powerful regulatory peptide of iron homeostasis, is mainly produced by hepatocytes and to a lesser extent by immune cells such as macrophages. Hepcidin binds to ferroportin inducing its internalization and degradation. Such interaction is primordial for regulating macrophage iron levels as well as systemic iron homeostasis.

Free haemoglobin and heme, two major constitutive proteins of erythrocytes, are then recognised and carried by haptoglobin and hemopexin proteins, respectively. CD163 and CD91 (the low-density lipoprotein receptor-related protein (LRP)/CD91) expressed on the surface of macrophages recognize and induce endocytosis of haptoglobin(Hp)/haemoglobin and heme/hemopexin complexes, respectively (Figure 5) [41]. After vacuolar Hb/Hp or heme/hemopexin complexes break down, heme is transported to the cytosol via HRG1 and then degraded by heme oxygenase 1 (HMOX1) [42] (Figure 5). HMOX1 degrades heme into biliverdin, carbon monoxide and iron [43]. Through this process, macrophages contribute to iron recycling and limit the pro-oxidant deleterious effects of free haemoglobin and heme in the circulation.

#### 2.3.2. Intracellular Haemolysis or Erythrocyte Phagocytosis

Extravascular haemolysis is defined as the most common mechanism for erythrocyte destruction and is mediated by tissue macrophages within the reticuloendothelial system [44]. This mechanism, named EP, consists of the recognition and engulfment of old or damaged erythrocytes by macrophages in the spleen, liver (Kupffer cells) and bone marrow. This specific phagocytosis of erythrocytes is crucial for the maintenance of homeostasis and for iron metabolism regulation via heme iron recycling. By removing senescent and damaged erythrocytes from the bloodstream, EP prevents the release of highly reactive components from erythrocytes, such as haemoglobin, heme and iron.

##### Physiological EP Process and Tissue Macrophages

The main physiological trigger of EP is eryptosis [45,46,47,48]. Eryptosis is a specific apoptotic mechanism for erythrocytes and is sometimes named “erythrocyte suicidal type of cell death”. This process can be triggered by several stimuli, such as oxidative stress, energy depletion and/or osmotic shock. Eryptosis includes different changes in erythrocytes, such as cell shrinkage, membrane blebbing and phosphatidylserine exposure at the outer membrane leaflet (membrane remodelling). Changes in the membrane and ion composition render erythrocytes denser. These characteristics allow the separation of old and young erythrocytes. Phospholipid asymmetry is also recognised as a key trigger for the recognition and extravascular removal of senescent erythrocytes by tissue macrophages [49,50].

The physiological destruction and clearance of eryptotic erythrocytes are mainly ensured by spleen macrophages. In this organ, erythrocytes are brought to vessels of different sizes, where they will encounter local splenic macrophages. In the spleen, erythrocytes will have to lengthen to move forward and pass through vessels of decreasing diameter. When erythrocytes are damaged or too rigid and lose their deformability, they are not able to pass through the interendothelial slits of the red pulp of the spleen. They will stay in this area and will be recognised by the red pulp macrophages, which are highly specialised, to clear erythrocytes from the bloodstream [51]. In fact, splenic macrophages are the main cells responsible for erythrocyte clearance via the EP process in physiological conditions [52]. However, as discussed before (Figure 4), when RBCs become damaged or present intrinsic defects that accelerate the process of eryptosis and shorten their life span, monocytes from the circulation play an important role in the clearance of erythrocytes by EP. The migration of such cells to the liver with their differentiation into macrophages indicates that the liver is a major site of pathological erythrocyte elimination and iron recycling [40]. Indeed, in a mouse model of haemolytic anaemia due to a mutation in erythroid pyruvate kinase, the accumulation of RBCs and iron in liver macrophages has been clearly observed [53,54].

One major aspect of EP by tissue macrophages is recycling of the heme iron present in large amounts in erythrocytes (Figure 2 and Figure 5). Thereby, macrophages play a key role in iron homeostasis in the recycling process for the production of new erythrocytes. Once in the phagocytic vacuole, the erythrocyte is digested by hydrolytic enzymes and ROS, leading to the release of haemoglobin and then heme within the vacuole (Figure 5). Heme is then transported through the phagosomal membrane via the HRG1 transporter to reach the cytosol [55,56]. HMOX1 degrades heme into biliverdin, carbon monoxide and iron [43]. Iron is then stored as ferritin (an iron storage protein) or according to the need is transported outside the cell via the only known iron exporter ferroportin (Fpn) [57,58]. Oxidases, such as soluble and GPI-membrane anchored ceruloplasmin, are also involved in this transport [59]. One major regulator of this iron transport is a small peptide named hepcidin (Hepc) [60]. Hepc, now known as the iron hormone, is mainly produced and secreted by not only hepatocytes in the liver but also to a lesser extent in other organs and cells, such as monocytes and macrophages [61,62]. To date, Fpn is the only known target for Hepc, and it was demonstrated that Hepc binds to Fpn, promoting the internalization and degradation of the transporter through an ubiquitination process [63,64,65,66]. Hepc regulation occurs at the transcriptional level, being upregulated by inflammation/infection and by high iron concentrations, while erythropoietic demand, hypoxia, anaemia and a low iron status suppress Hepc expression [67,68].

##### Erythrocyte Binding and Erythrophagocytosis by Non-Specialised Cells

In the liver, resident macrophages (Kupffer cells) are not the only ones responsible for erythrocyte clearance. In fact, in 2011, by transfusing damaged erythrocyte-FITC labelled to macrophage-depleted mice, Lee et al., evidenced that erythrocytes are still sequestered in the hepatic sinusoid, suggesting that a population of liver cells other than Kupffer cells is also responsible for the erythrocyte sequestration mediated by PS exposure. These cells seem to be human hepatic sinusoidal endothelial cells (HSECs), which can bind damaged erythrocytes but are not able to engulf them. HSECs are not responsible for erythrocyte engulfment, but they participate in the clearance process. Indeed, HSECs enhance EP by presenting damaged erythrocytes to macrophages [69].

Mast cells are involved in adaptive and innate defences against pathogens by the secretion and release of pro-inflammatory molecules. In certain circumstances, mast cells can behave as professional phagocytes for the clearance of pathogens and micromolecular or particulate molecules, in addition to the secretion of molecular mediators [70]. Recently, mast cells were shown to exhibit phagocytic capacity for oxidatively damaged erythrocytes but not for normal erythrocytes in vitro and in vivo. In addition, such specific uptake of damaged erythrocytes was further enhanced when mast cells were in an activated state [71].

Endothelial cells have been reported to interact with damaged and oxidised erythrocytes. Recently, it has been evidenced that endothelial cell interaction with aged or diabetic erythrocytes is followed by EP [72]. Thus, in an atherothrombotic environment, endothelial cells may act in erythrocyte clearance. The abnormal adhesion of erythrocytes to the endothelium is thought to be implicated in vascular occlusion and endothelial cell dysfunction.

Vascular smooth muscle cells (VSMCs) are the main cell type present in the early intimal thickening of most stages of human ATH [73]. They are capable of phagocytosis of microparticles and senescent cells. They are able to bind to and engulf senescent cells, including senescent erythrocytes in the atheroma environment in the earliest stages [74,75]. This EP could contribute to lipid and iron accumulation, as well as foam cell formation [76]. This recognition seems to occur via a functional phosphatidylserine receptor on smooth muscle cells [77].

## 3. Atherosclerosis, a Pathology in Three Steps

Cardiovascular disease represents the leading cause of mortality, accounting for 17.5 million deaths in 2012 (Cardiovascular diseases Fact Sheet (reviewed September 2016), WHO). Cardiovascular disease is mainly associated with the development of ATH in the arterial wall of medium and large arteries. Stenosis and occlusion lead to ischaemia of downstream tissues, causing myocardial infarction or stroke. ATH is a complex pathology associated with well-recognised risk factors, such as hyperglycaemia, high blood pressure and dyslipidaemia, including high concentrations of circulating low-density lipoprotein (LDL) [78]. Specific detrimental environmental conditions, such as sedentary lifestyle, rich food intake or tobacco smoking also contribute to atherosclerotic plaque formation.

### 3.1. Fatty Streak Development

Atherosclerotic plaques preferentially develop at specific sites of the arterial tree, such as inner curves and bifurcations where blood flow becomes disturbed and low endothelial shear stress occurs [79,80]. At these specific sites, the endothelium’s physiology is perturbed. In response to blood flow disturbance, endothelium synthesizes specific factors, such as nitric oxide (NO) or endothelin-1, to adapt the vessel diameter to these impaired blood circulating conditions. The increased arterial inner wall thickness is named “adaptive intimal thickening” [81]. Plaque formation begins at the site of endothelial perturbations, leading to lesions or erosions that favour the entry and accumulation of circulating LDL particles into the intimal space. LDL is highly atherogenic and rich in cholesterol. LDL particles enter the intimal space either via interactions with LDL receptors at the endothelial surface of the cell and subsequent transcytosis (apical endocytosis and basal exocytosis) or by paracellular passage favoured by a high circulating concentration. The accumulation of LDL particles in the intimal space is increased due to their high affinity for extracellular matrix proteoglycans. The extracellular LDL particle is modified by oxidation, glycosylation and/or enzymatic proteolysis. The endothelium is locally activated, and its unbalanced redox status is exacerbated. Furthermore, endothelial cells synthesize adhesion molecules, such as vascular cell adhesion molecule 1 (VCAM-1) or intercellular adhesion molecule 1 (ICAM-1), at their surface to recruit immune cells, such as monocytes [82]. Native and modified LDL particles are taken up by macrophages or SMCs. Activated macrophages may enhance inflammation and redox unbalanced states by producing cytokines and ROS. Endocytosed LDL particles release their cholesterol content into macrophages or SMCs, where they accumulate and form lipid droplets. This excess of lipids transforms macrophages and SMCs into foam cells that accumulate in the intimal space to form fatty streaks [83]. Cytokines and growth factors, locally secreted by activated endothelial cells, platelets and inflammatory cells (endothelin-1, thrombin and interferon-γ), stimulate SMC proliferation and migration from the media to the intimal space. Once there, they produce extracellular matrix components, such as collagen fibres forming a fibrous cap [84,85].

### 3.2. Fibrous Cap Formation

The newly formed fibrous cap “sequesters/isolates” the lipid core composed of macrophages and SMC foam cells from the blood flow. The pro-inflammatory and pro-oxidant environment induces cell death by necrosis and/or apoptosis. The lipid core is transformed into a necrotic core with dead cells releasing their cellular components, including lipid droplets [83]. The thickened fibrous cap and necrotic core lead to an increase in the stenosis of the blood vessel. At this stage, the atherosclerotic plaque is generally considered as stable, with limited risk of rupture. However, the intimal thickening characterised by an accumulation of cells, lipids and matrix causes an insufficient O_2_ supply, generating hypoxia [86]. This local hypoxia, associated with an inflammatory process, stimulates the formation of new vessels originating from adventitial vasa vasora towards the atherosclerotic plaque [87]. Hypoxia induces the expression of hypoxia inducible factor 1 alpha (HIF-1α), a transcription factor involved in the initiation of angiogenesis in atherosclerotic plaques [88]. Endothelial cell growth is stimulated by vascular endothelial growth factor A (VEGF-A) via its receptor vascular endothelial growth factor receptor 2 (VEGFR-2). Then, E26 transformation specific 1 (ETS-1) acts as a transcription factor and induces VEGF expression [89]. ETS-1 also induces metalloproteinase (MMP) synthesis, such as MMP-1, which participates in the degradation of the junction ECM between endothelial cells [90]. Furthermore, it induces endothelial cell migration for neoangiogenesis [90]. The new vessels formed in atherosclerotic plaques are fragile, immature and leaky; participate in intraplaque haemorrhage (IPH); and can trigger plaque rupture [87]. ROS produced in the necrotic core can be mediators and contribute to the neovascularization. Indeed, ROS stimulate the expression of VEGF in endothelial cells [91].

### 3.3. Atherosclerotic Plaque Complications

Atherosclerotic plaque can develop in different sizes and compositions. These plaques can become prone to rupture (“vulnerable plaques”) and are characterised by the presence of neovascularization, intraplaque haemorrhage, calcification and a thin fibrous cap.

These events involve different actors, among which are the erythrocytes that will play a role in the destabilization and rupture of the atherosclerotic plaque that participates in the formation of a thrombus, causing a vascular accident.

## 4. Involvement of Erythrocytes in the Progression to Atherothrombosis

### 4.1. Erythrocytes Can Contribute to ATH Progression and Complications

#### 4.1.1. Intraplaque Haemorrhage

**Figure 6:**
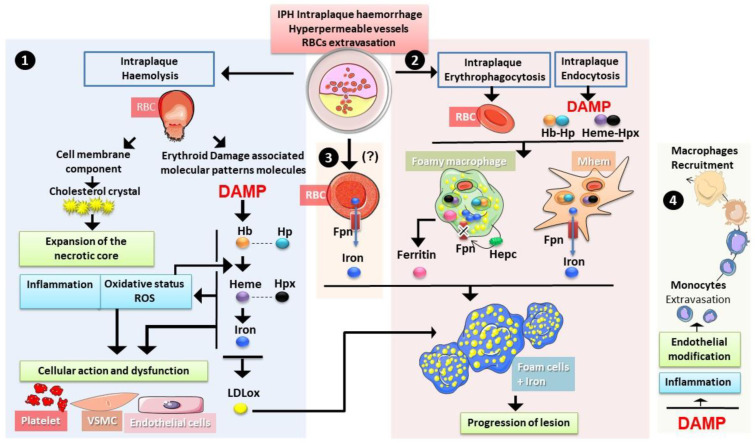
Erythrocytes in intraplaque haemorrhage. During intraplaque haemolysis **1**. and rupture lysis, RBCs liberate both cholesterol and erythroid damage-associated signalling molecule patterns ((DAMPs) haemoglobin Hb, heme and iron), both contributing to the expansion of the necrotic core and to the increase in oxidative stress and inflammation. Such changes could have an impact on the function of endothelial cells, vascular smooth muscle cells (VSMC) and platelets. Haemoglobin and iron can promote the oxidation of LDL particles accelerating the foam cell differentiation. Erythrophagocytosis as well as endocytosis of heme/hemopexin and haemoglobin/haptoglobin complexes **2**. also occurs in the plaque with an important role of macrophages. The haemorrhage-associated macrophages (Mhem) are prone to recycling iron from both erythrophagocytosis and heme containing-complexes endocytosis, whereas foamy macrophages (Mox) tend to accumulate iron with lipids. Autocrine expression of hepcidin (Hepc) seems to play a role in iron retention. Ferritin secretion by these cells has been reported. Another source of iron could exist directly from erythrocytes via the expression of ferroportin (Fpn) **3**. Both iron and ferritin present in the plaque with oxidised low-density lipoprotein (oxLDL) could trigger the formation of foam cells, promoting the progression of the lesion and the instability of the plaque. In addition, **4**. erythroid DAMPs were shown to increase expression of adhesion molecules by endothelial cells and to have proinflammatory effects. Both phenomena contribute to the recruitment of more monocytes/macrophages in the plaque.

IPHs deliver a large amount of erythrocytes into the atherosclerotic plaque. The pro-inflammatory and pro-oxidative environment of the necrotic core induces rapid damage to erythrocytes, making them more vulnerable to haemolysis or phagocytosis by intraplaque macrophages and smooth muscle cells. Both processes of haemolysis (rupture lysis and EP) could occur within the atheromatous plaque (Figure 6). Such a phenomenon can induce the release in the necrotic core of some erythrocyte components, such as cholesterol and erythroid damage-associated signalling molecule patterns (DAMPs). DAMPs include iron, heme and haemoglobin molecules with numerous effects on the homeostasis of the plaque. Thereby, erythrocytes contribute to necrotic core expansion. Erythrocyte products also contribute to increased oxidative stress via enhanced lipid oxidation and increased ROS production.

#### 4.1.2. Erythrocytes, a Source of Cholesterol That Fuels Plaque Development and Complications

Erythrocytes are predominant in IPH [92], and their extravasation plays many roles in plaque progression, complication and rupture. The proportion of cholesterol in the membranes of erythrocytes is strongly dependent on circulating lipoprotein concentrations [93]. Indeed, a balance is established between the total cholesterol in the membrane of the erythrocyte and plasma cholesterol concentration [94]. Therefore, erythrocyte cholesterol is involved in necrotic core expansion (Figure 6). In Kolodgie’s work, autologous erythrocytes were injected at the atherosclerotic lesion site in the abdominal aorta of rabbits fed with or without a proatherogenic diet [95]. It was shown that an injection of 25 to 50 µL of autologous erythrocytes induced an important increase in lipids associated with the formation of cholesterol crystals into the atherosclerotic lesions of rabbits fed a proatherogenic diet compared to control rabbits. Furthermore, Virmani et al. estimated the contribution of erythrocyte membrane cholesterol to necrotic core expansion [96]. They showed that 100 µL of erythrocytes at 50% haematocrit containing 10% cholesterol could contribute to enlarging the necrotic core by more than 0.2 mm^3^. This evaluation represents a bleeding volume of 0.137 µL of whole blood per day for 2 years of successive IPHs. According to these studies, the contribution of erythrocyte cholesterol to necrotic core volume appears substantial.

#### 4.1.3. Erythrocytes in the Plaque: Source of Damage-Associated Molecular Pattern with Pathological Consequences

Bleeding in the atherosclerotic plaque exposes erythrocytes to lipid peroxides or oxidation products and induces erythrocyte lysis and EP. The products of erythrocyte haemolysis can be recognised as damage-associated molecular patterns (DAMPs), promoting chronic inflammation and oxidative stress [97].

#### Haemoglobin and Heme

In a physiologic context, the erythrocyte content released following haemolysis is handled by specific proteins, such as haptoglobin and hemopexin (see Section 2.3.1). In cthe ase of massive haemolysis in the circulation, haptoglobin and hemopexin can be overwhelmed. Therefore, free haemoglobin and heme will accumulate in plasma and potentially cause cell damage [98].

**Figure 7:**
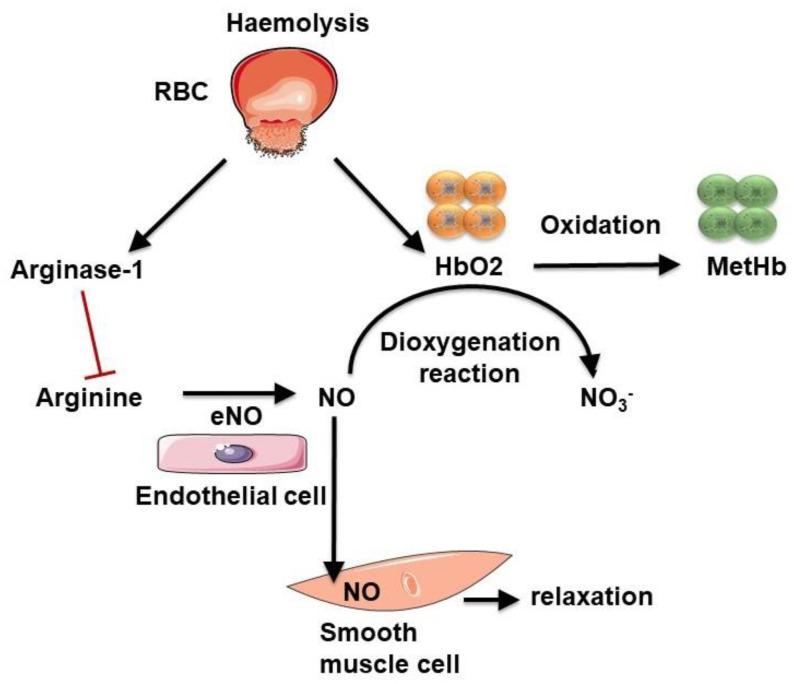
Influence of haemolysis on nitric oxide (NO) bioavailability. Intravascular haemolysis induces arginase-1 and haemoglobin release. Arginase-1 produces ornithine from arginine, the required substrate for NO production. Oxygenated haemoglobin (HbO2) can scavenge NO by a dioxygenation reaction, producing inert nitrate and methemoglobin (MetHb). These two reactions decrease NO bioavailability.

Free plasma haemoglobin may act as a strong NO scavenger, reducing its bioavailability and thus vasodilatation. NO is an important signalling molecule that regulates vasoactivity, blood flow and haemorheology [99,100]. The reaction producing NO is catalysed by nitric oxide synthase from arginine and O_2_ (Figure 7). Once produced, NO diffuses and enters the intraluminal space to act on VSMCs via the activation of guanylate cyclase, inducing their relaxation and thus vasorelaxation. Erythrocytes can affect vascular function. Indeed, O_2_ pressure regulates the O_2_ release (by haemoglobin) required by endothelial nitric oxide synthase (eNOS) to produce NO [101]. Haemoglobin present within erythrocytes also acts as a NO scavenger, reducing its bioavailability. Outside the cell, after haemolysis, haemoglobin released into the bloodstream scavenges NO faster than that contained in intact erythrocytes [102]. Oxygenated haemoglobin (ferroHb also referred to as HbO_2_), through a dioxygenation reaction, reacts with NO to produce methemoglobin and inert nitrate [102,103]. All these mechanisms decrease NO availability.

During haemolysis, erythrocyte releases not only haemoglobin but also arginase I, which converts l-arginine into ornithine, reducing the bioavailability of arginine, which is the substrate for NO production. Indeed, a recent study showed that erythrocytes from diabetic patients induced endothelial cell dysfunction, which can be attenuated when inhibiting erythrocyte arginase I (along with ROS scavenging), suggesting that erythrocytes are important in the development of endothelial dysfunction [104].

If not neutralised, free haemoglobin can be damaging to cells. Indeed, it can react with oxidants, such as hydrogen peroxide, which gives rise to oxidised haemoglobin and the subsequent release of heme. Heme, through the presence of iron, can also oxidise other proteins, inducing the production of reactive species, and, therefore, oxidative stress [105]. Free iron, which is considered a powerful pro-oxidant, can also cause severe oxidative stress through the Fenton reaction and generate reactive molecules. Furthermore, free haemoglobin and heme, by their pro-oxidative properties, can promote LDL oxidation to mainly produce 5-hydroxy-2-amino valeric acid (HAVA) and products toxic to the endothelium [106,107]. The hydrophobicity of heme favours its association with the lipid moiety of the plaque and thus enhances the lipid-associated oxidative stress mediated by iron [107]. Derivative forms of haemoglobin (metHb and ferrylHb) can initiate oxidative modifications of LDL particles [108]. Nagy et al., have shown that, following exposure of atherosclerotic plaque to free heme, the lipid moiety of LDL particles can be oxidised. Moreover, these lipids oxidised by heme are highly toxic to endothelial cells [109]. LDL oxidation by heme amplifies the pro-oxidant environment of the atherosclerotic plaque. Moreover, high levels of oxidised LDL particles and HAVA have been detected in diabetic subjects, resulting from high amounts of heme [107]. Oxidised LDL particles from the atheromatous plaque can also induce oxidative modifications of haemoglobin, leading to the production of ferrylHb [108]. It has been shown that ferrylHb exerts pro-inflammatory and cytotoxic effects on the vascular endothelium, promoting its permeability and monocyte adhesion [110].

#### Iron

Evidence for the presence of catalytic iron in human atherosclerotic lesions was previously provided by Smith et al., who reported lipid peroxidation induced by the iron-rich content of the plaque [111]. Pang et al. reported that both h- and l-ferritin (iron storage proteins) are highly expressed in human atherosclerotic plaques, being induced on both endothelial cells and macrophages at the early onset of the lesion and prior to the occurrence of the IPH often observed in advanced lesions [112]. Many observations suggest that the origin of pathological iron deposition in atheromatous plaques is linked to erythrocytes and macrophages, with signs of EP, haemoglobin catabolism and strong ferritin accumulation in these cells [95,113,114,115].

During IPH, the massive erythrocyte extravasation contributes greatly to iron accumulation in the plaque (Figure 6). Indeed, it has been shown that, in human carotid plaques, an important accumulation of erythrocyte markers, such as glycophorin A, was associated with increased iron and macrophages and was correlated with larger necrotic cores [95]. Both haemolysis and elimination of erythrocytes by macrophages occur in the plaque’s environment (Figure 6). Through their high capacity to capture heme iron from EP or from haemoglobin and heme endocytosis, different populations of intraplaque macrophages play a key role in this process.

A novel polarised phenotype, first called haemorrhage-associated macrophages (HA macs) and later renamed as Mhem macrophages, was identified by Boyle et al., in human plaques with fatal coronary thrombosis [116]. In parallel, a macrophage phenotype named M(Hb) and driven by Hb-Hp during macrophage differentiation was reported, corresponding to the Mhem phenotype described above [117]. These HA-macs (Mhem or M(Hb)) are localised around the haematoma, being mostly absent in stable and haemorrhage-free plaques, and express high levels of HMOX1 and CD163, the Hb-Hp scavenger receptor [117]. This polarised phenotype was differentiated in vitro by exposure of macrophages to oxidised erythrocytes, Hb or heme for 7 days and was accompanied by HMOX1 upregulation in an Nrf2-dependent pathway [118]. Interestingly, Mhem and M(Hb) are distinct from lipid-laden macrophages (foam cells), presenting low lipid content. The upregulation of apoE, as well as cholesterol exporters (ABCA1 and ABCG1), promote cholesterol efflux [116], thereby preventing these cells from differentiating into foam cells. These cells are considered to be anti-atherogenic, despite their probable contribution to increased iron levels in the plaque. Increased iron exporter Fpn levels and subsequent increased iron export in M(Hb) macrophages may result in low intracellular iron and low ROS [117].

On the other hand, iron accumulation in other macrophage populations (not Mhem), such as foam cells, during plaque formation and haemorrhage seems to be an important risk factor for plaque destabilization. Indeed an “iron hypothesis”, as a modifiable risk factor for ATH [119,120], was proposed 30 years ago by Sullivan. Sullivan suggested that high local iron concentrations would enhance the availability of redox-active iron at the site of oxidative and/or inflammatory injuries, where iron could locally play a role in the progression of atherosclerotic lesions. Accordingly, a state of sustained iron depletion or mild iron deficiency induced by regular phlebotomy or blood loss associated with menstruation had a protective effect against the progression of ATH [120,121,122]. Many observations suggest that iron depletion decreased atherosclerotic lesions and delayed the onset of the disease and could thus be considered as protective towards the development of vascular diseases [123,124,125,126].

A common argument pointed out against the “iron hypothesis” relies on the lack of association between the iron overload disease hemochromatosis (HH) and an increased susceptibility to the development of ATH [127,128]. In response to the controversy around HH, a refinement of the “iron hypothesis” was later presented by Sullivan [129,130,131]. HH is characterised by systemic iron overload as result of a disrupted Hepc-Fpn axis, causing continuous iron absorption and mobilization from body stores. On the other hand, patients with HH present iron-depleted macrophages as a result of inappropriately low levels of Hepc or Fpn gain-of-function mutations. Sullivan proposed that macrophage iron retention could be an essential feature for the progression of lesions in ATH.

In support of the “iron hypothesis”, several observations have pointed out that Hepc-dependent iron retention in intraplaque macrophages likely contribute to the formation of foam cells and subsequent progression of the lesion [132]. Pharmacological suppression of Hepc in apoE-deficient mice using a bone morphogenetic protein (BMP) inhibitor decreased macrophage iron content and increased cholesterol efflux [133]. This resulted in reduced foam cell formation and decreased lipid burden and atherosclerotic lesions, pointing to possible protection against the progression of ATH via Hepc inhibition [133]. Accordingly, overexpressed Hepc production in carotid arteries affected the plaque’s composition, increasing the number of intraplaque macrophages with oxidised low-density lipoprotein (oxLDL) and iron retention, as well as increasing oxidative stress and production of pro-inflammatory cytokines [134]. In Hamp^−/−^/ Ldlr^−/−^ mice, a hyperlipidaemic mouse model with Hepc deficiency, Malhotra et al. observed decreased macrophage iron, reduced aortic macrophage inflammatory phenotype and protection from ATH [135].

In addition to the role of systemic Hepc produced by hepatocytes, autocrine expression of Hepc in plaque macrophage foam cells was suggested. In vitro, Mox macrophages differentiated upon exposure to oxidised phospholipids [136] were shown to expressed Hepc [137,138], suggesting that a microenvironment rich in oxLDL and pro-inflammatory cytokines could promote macrophage iron retention and lipid accumulation. Importantly, using the mouse atherogenic model, ApoE^−/−^ mice with a macrophage-specific Fpn deficiency (ApoE^−/−^Fpn1 ^LysM/LysM^), Cai et al. demonstrated that Fpn deficiency and, therefore, iron overload in macrophages dramatically accelerated the progression of ATH in mice. Noteworthy, iron loading in macrophages was reported to favour cholesterol uptake and accumulation by upregulating scavenger receptors (MSR1) [139]. In addition, cholesterol exporters (ABCA1 and ABCG1) were shown to be repressed in macrophages with iron overload [140]. Overall, the interaction among Hepc, macrophage iron retention and lipid accumulation is critical for the development of foam cells, leading to plaque formation and potentially destabilization.

In addition to macrophages, it has been observed that erythrocytes, which contain large amounts of iron for haemoglobin synthesis, express Fpn [141,142,143,144]. The erythroid expression of the iron exporter seems to be critical for systemic iron homeostasis, with possible redistribution of iron to tissue cells in the case of systemic iron deficiency [58,142]. Interestingly, the concentration of iron in the circulation was reduced by 20% in mice disabled for Slc40a1 (Fpn) gene expression at the erythroblastic level [142]. Fpn, by exporting the free iron derived from the auto-oxidation of haemoglobin, could also protect erythrocytes from oxidative stress [142,144]. Therefore, it is tempting to speculate that erythrocytes entering the intraplaque environment contribute directly to an increase in extracellular iron via its efflux by Fpn (Figure 6). Investigating, in more detail, the expression of Fpn in intraplaque erythrocytes, as well as in modified (glycation) erythrocytes (see Section 4.2), would be of great interest to clarify the role of erythrocyte-derived iron in atherogenesis.

After the erythrocytes enter the plaque, the presence of haemoglobin, heme and iron can trigger deleterious effects on atheromatous cells by modulating oxidative stress and inflammation (Figure 6) [132,145,146]. As described before, the presence of catalytic iron in the intraplaque space can promote LDL oxidation, with their accumulation in macrophages and foam cell formation [147]. Haemoglobin, heme and iron have also been shown to induce other proatherogenic processes, such as endothelial activation, smooth muscle cell proliferation and migration, platelet aggregation and macrophage activation [132,145,146]. A consequence of heme- and iron-induced pro-inflammatory effects with increased expression of adhesion molecules in endothelial cells is the increased recruitment of monocytes into the plaque.

### 4.2. Enhanced Proatherothrombotic Capacity of Glycated Erythrocytes

#### 4.2.1. Erythrocyte Protein Glycation

**Figure 8:**
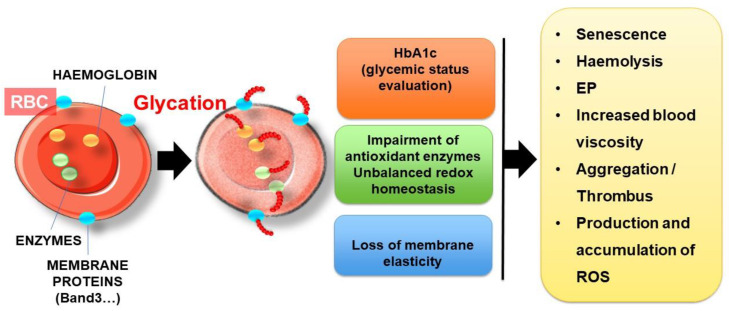
Deleterious effects of erythrocyte glycation. Hyperglycaemia causes glycation of many RBC components, such as haemoglobin, antioxidant enzymes and cell surface membrane proteins. This phenomenon induces unbalanced redox homeostasis with the loss of membrane elasticity. As a consequence, erythrocyte senescence, haemolysis, EP, erythrocyte aggregation and ROS production are enhanced.

Whether in the systemic circulation or in the adventitial vasa vasora of atherosclerotic plaques, erythrocytes are continuously exposed to glucose and are highly sensitive to glycation. In hyperglycaemic situations, the glycation process could affect most erythrocyte proteins, such as haemoglobin and cytoskeletal proteins and enzymes (Figure 8).

Glycation is a long process affecting mainly long-lived proteins in the circulation, such as albumin and constitutive proteins of erythrocytes. Glycation is a slow non-enzymatic reaction involving reducing sugar (glucose or derivatives) and the free amino groups of proteins, reversibly forming a Schiff base product. Following molecular rearrangements, this unstable intermediary product evolves gradually into Amadori products, such as ketoamine. The latter could undergo further modification (rearrangement, oxidation, polymerization and cleavage) to give rise to irreversible advanced glycation end products [148]. Reducing sugars, such as glucose, are not the only factor responsible for glycation. Some reactive α-oxoaldehyde derivatives produced during the glycolysis of glucose oxidation, such as MGO, are highly reactive and can also rapidly and spontaneously react with free amino groups, eventually producing advanced glycation end products (AGEs) [149].

##### Haemoglobin

Like other long-lived circulating proteins directly exposed to numerous deleterious metabolites, haemoglobin is highly sensitive to glycoxidative processes. Indeed, the passive transfer of glucose into erythrocytes is mediated by the insulin-dependent glucose transporter GLUT-1. This glucose transporter facilitates the cellular uptake of glucose and allows the equilibrium between glucose concentration in erythrocyte cytosol and in plasma. In hyperglycaemic conditions [150,151], glycated haemoglobin (HbA1c) is considered as the gold standard for the evaluation of glycaemic status and therapeutic efficacy [152,153].

Except for diseases associated with erythrocyte disorders (e.g., sickle cell disease and thalassaemia), HbA1c represents a relevant indicator of blood glucose exposure over a 2-3-month period [148]. In HbA1c, glucose is bound to the *N*-terminal valine residue of each β-chain of the haemoglobin molecule [154]. Haemoglobin is the major protein of RBCs (95%). The mechanism of haemoglobin glycation has been extensively studied, and many in vitro experiments have shown that glucose can also be attached to other amino acids, such as lysine, and in either the α or β subunits of haemoglobin [155,156,157]. This has been confirmed by several studies conducted in vivo in diabetic patients, focusing on the characterisation of glycated proteins in erythrocytes, aimed at determining the specific glycation sites [158,159]. For instance, the number of glycation sites in haemoglobin chains was found to be correlated to the percentage of HbA1c [158].

##### Membrane Proteins

Apart from haemoglobin, many other erythrocyte proteins were found to be glycated in diabetic patients, in particular, membrane proteins, such as ankyrin, spectrin, protein 4, band.3 or glycophorin A [158,159,160]. Many glycated proteins were effectively identified in erythrocytes from diabetics, and both cytosolic and membrane proteins were found to be affected [159,161]. These glycated proteins result in the progressive and irreversible formation of advanced glycation end products (AGEs), whose accumulation in plasma is strongly linked to diabetic complications [162]. Amongst AGE adducts, which could be formed on the surface of diabetic erythrocytes, pentosidine, carboxy -methyllysine (CML), carboxyethyllysine (CEL) and Arg-pyrimidine (Arg-P) have been reported to be the most predominant in vivo in diabetics [163,164]. An in vitro study confirmed the formation of CML and CEL as the main AGE adducts in erythrocytes, with accumulation in a glucose-dependent manner [165]. In vivo, CML can give rise to the formation of highly reactive and deleterious dicarbonyl compounds. This dominant circulation AGE affects the main membrane proteins and can accumulate within erythrocytes during their lifespan [166].

The glycoxidation process, which is known to be exacerbated in diabetic situations, could affect both haemoglobin and the main membrane proteins of RBCs. To understand this key role in erythrocyte disturbance, numerous in vitro studies have focused on the effect of glycation on the integrity, functionality and metabolism of erythrocytes. Due to the low speed of the reaction (up to several weeks), in vitro glycation of erythrocytes is usually performed by exposure to high glucose concentrations (between 30 and 100 mM) for a short period of time (between 24 and 120 h) [167,168,169]. These in vitro models are often used to investigate erythrocyte modifications in diabetes [72,170].

These in vitro studies showed globally that AGE accumulation and the resulting excessive ROS production leads to oxidative damage and structural/functional alterations of the proteins and membrane lipids of erythrocytes. Due to their fundamental role in O_2_ transport and CO_2_ elimination, erythrocyte membrane components (proteins and lipids) are particularly sensitive to oxidation, which can impair their structure and function, such as Na^+^/K^+^-ATPase and Ca^2+^-ATPase activities [171,172,173]. The Ca^2+^-ATPase pump enables the active transport of Ca^2+^ across the erythrocyte’s cell membrane in order to maintain a low intracellular calcium content. With the Na^+^/K^+^-ATPase pump, they participate in the ion homeostasis of blood cells by regulating cell volume and nutrient uptake [173]. In vitro glycation with glucose clearly affects pump activities, causing cytosolic calcium and potassium ion accumulation in erythrocytes [171,172,173,174].

##### Erythrocyte Enzymes

Apart from enzymes responsible for ion transport through the permeable cation channels, other erythrocyte enzymes could be dramatically impaired upon in vitro or in vivo glycation. For instance, the redox balance regulating enzymes, such as catalase, glutathione S-transferase and glutathione reductase, were found to be impaired in erythrocytes after exposure to high concentration of glucose (100 mM) for a long time (above 72 h) [172]. Similarly, the percentage of the glycated form of Cu, Zn-SOD (superoxide dismutase), which has been shown to be inactive, was found to be increased in the erythrocytes of diabetic patients [175]. These enzymes are the first line of defence against ROS and reactive nitrogen species (RNS), and their alteration directly impacts the intracellular antioxidant potential of erythrocytes by increasing the GSSG/GSH ratio. The glutathione (GSH) of erythrocytes is considered the most abundant circulating antioxidant pool in the body [176]. Reduced glutathione could neutralize superoxide products that give rise to the formation of oxidation products, including glutathione disulphide (GSSG). It can also reduce hydrogen peroxide and lipid peroxides via glutathione peroxidase.

Although many clinical studies in diabetic patients seem to show conflicting results regarding the impact of hyperglycaemia on the GSH concentration of erythrocytes [16], some of them showed that glycation and oxidation can damage to enzymes involved in GSH synthesis [177,178,179]. The protective effects of erythrocytes against ROS-induced oxidative damage are mainly due to catalase and glutathione metabolism, which are impaired in glycoxidative situations.

Alteration of the main antioxidant enzymes associated with unbalanced redox homeostasis exacerbates intracellular ROS production in erythrocytes in a glycoxidative context [72,170,180]. As the main provider of cellular O_2_^•−^, NADPH oxidase activity was found to be strongly correlated with HbA1c in diabetic patients with cataracts, suggesting the effect of the glycaemic status on the pro-oxidant enzyme [181].

#### 4.2.2. Consequences of Glycation: How Erythrocyte Glycation Is Involved in Atherothrombosis

**Figure 9:**
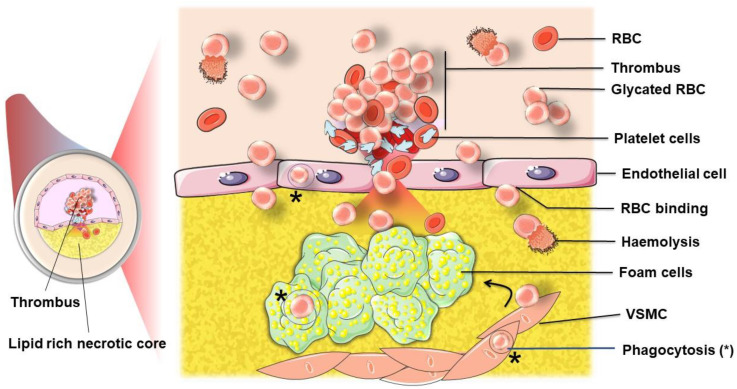
Participation of RBCs to atherothrombosis. Following plaque rupture, RBCs can further infiltrate the plaque where they are either phagocytosed (*) by endothelial cells, macrophages and vascular smooth muscle cells (VSMC), or lysed. Erythrocytes, by their adhesive and aggregation properties, exacerbated when glycated, also contribute, with platelets, to the thrombus formation. When glycated or aged, phosphatidylserine exposing erythrocytes can further interact with activated platelets [182]. This process participates in the occlusive phenomenon.

In ATH, glycated erythrocytes can be involved not only in neovessel leakage and intraplaque haemorrhage but also in the vessel lumen with thrombus formation following plaque rupture (Figure 9). Glycation impacts several properties and functions of erythrocytes, as developed below.

##### Loss of Deformability and Aggregability

According to several clinical studies, an inverse correlation was shown between HbA1c and red blood cell deformability in diabetic patients [183,184]. This partial loss of membrane elasticity associated with an increased viscosity in diabetic patients has been suggested to be the consequence of glycation-induced modifications in the membranes of erythrocytes [185]. Closely related to the glycation phenomenon, the effects of oxidative conditions on erythrocyte homeostasis could directly affect the efficacy of membrane band.3 protein in its role as an anion exchanger, ensuring ion balance, tissue oxygenation and erythrocyte deformability [186]. Similarly, one of the essential proteins contributing to erythrocyte flexibility, β-actin, can potentially be glycated upon exposure to high glucose concentrations, leading to a decrease in erythrocyte deformability [187]. In contrast, another key protein, spectrin, could not be glycated without the prior translocation of phosphatidylserine (PS) from the inner to the outer lipid leaflet. This protective mechanism, limiting the glycation of spectrin, is mediated by ATP-driven phospholipid translocase (flippase) and allows the maintenance of critical membrane function for erythrocytes [151].

Denaturation of the membranes of erythrocytes upon the effects of glycation and oxidative stress induces abnormal viscoelastic properties, as well as an increased aggregation of RBCs, commonly encountered in diabetic patients [188,189,190]. Several clinical studies have suggested that increased erythrocyte aggregation is directly associated with vascular complications of diabetes [191,192]. In diabetes, glycation and oxidative stress induce changes in the composition of the erythrocyte’s membrane, resulting in a reduction in the negative charges carried by the sialic acid of glycoproteins. These negative charges contribute to the electrostatic repulsion between red blood cells and consequently limit their aggregation [193]. In parallel, the high level of plasma fibrinogen found in diabetic patients associated with the loss of erythrocyte deformability can act synergistically on their aggregability [194,195]. Consistent with decreased membrane flexibility, diabetes, through the molecular mechanism of glycation and oxidative stress, is associated with increased erythrocyte aggregability and fragility and decreased survival [196,197,198].

Aggregation and disaggregation are natural and reversible processes resulting from a balance between factors promoting aggregation (fibrinogen and surface charge density) and factors preventing aggregation (albumin). Hyperaggregation of erythrocytes is usually attributed to a reduction in negative charges in the membrane, a reduction in fibrinogen plasma levels and a decrease in albumin. Diabetes has been reported to increase erythrocyte aggregability with a reduction in negative charges, elevated fibrinogen and decreased albumin plasma levels [188,199]. Furthermore, a significant correlation between HbA1c and erythrocyte aggregation was evidenced [200]. Erythrocyte aggregation plays an important role in the pathophysiology of blood flow and is considered the main determinant for diabetic complications [201]. Indeed, the hyperaggregation of erythrocytes impairs blood flow and induces haemodynamic perturbations. Erythrocyte aggregates can form in a zone of low shear forces.

Such changes in haemorheologic and haemodynamic factors could play an important role in cardiovascular diseases. A loss of deformability and increased adhesion could especially initiate or aggravate plaque rupture and thrombus formation [202].

##### Senescence and Haemolysis of Glycated Erythrocytes

Prolonged exposure to high glucose concentrations and glycation affects the membrane fluidity of erythrocytes, increasing their rigidity and reducing their elasticity as described above. Many studies have shown that the increased haemolysis of glycated erythrocytes is associated with this loss of elasticity [170,203]. In addition, increased oxidative damage and reduced antioxidant defences lead to an accelerated senescence process in erythrocytes [204]. Moreover, in vitro glycation with glucose and oxidative stress clearly alters pump activity, causing cytosolic calcium and potassium ion accumulation in erythrocytes [171,172,173,174]. The intracellular increase in calcium resulting from the alteration of ion transport is one of the main elements that trigger eryptosis [205]. Indeed, it has been shown that oxidative stress and exposure to excess glucose concentrations could activate the Ca^2+^ pump, inducing a massive entry of calcium [171]. This rise in intracellular calcium triggers cell shrinkage, induces scramblase and thus phosphatidylserine exposure. High intracellular calcium concentrations also induce an efflux of K^+^, causing the hyperpolarization of the membranes of erythrocytes. The depletion in Cl^−^ and K^+^ induces the shrinkage of erythrocytes due to a decrease in KCl concentration [47].

**Figure 10:**
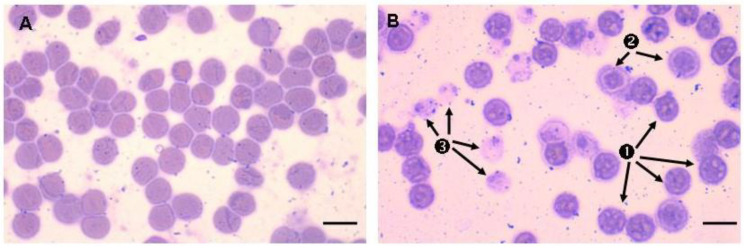
Glycation induces senescence and haemolysis of erythrocytes. Erythrocyte smears stained with May-Grünwald Giemsa stain for (**A**) normal erythrocytes. There is no porous structure in haemoglobin repartition in non-glycated erythrocytes. (**B**) Glycated erythrocytes incubated with 50 mM of glucose for 5 days at 37 °C display different morphologies. Arrows 1 feature erythrocytes with non-homogeneous structure and repartition of haemoglobin with presence of vacuoles. Arrows 2 feature erythrocytes with more condensed haemoglobin and a peripheral clear area. Arrows 3 feature erythrocyte ghosts corresponding to senescent RBCs after the release of haemoglobin following haemolysis. Bars indicate 10 μm.

More recently, several studies have shown that the in vitro glycation of erythrocytes with glucose or derivatives (such as methylglyoxal) could induce phosphatidylserine exposure at their membranes, which is a signal of senescence preceding haemolysis [72,170,180]. Following in vitro glycation, erythrocytes display heterogeneous and abnormal morphology, which is characteristic of senescent cells: contracted cells with a smaller size in which haemoglobin appears condensed and irregularly distributed inside erythrocytes. “Ghost” erythrocytes have also been described, indicating that many cells have totally lost their haemoglobin content due to oxidative haemolysis (Figure 10).

##### Clearance and Erythrophagocytosis (EP)

**Figure 11:**
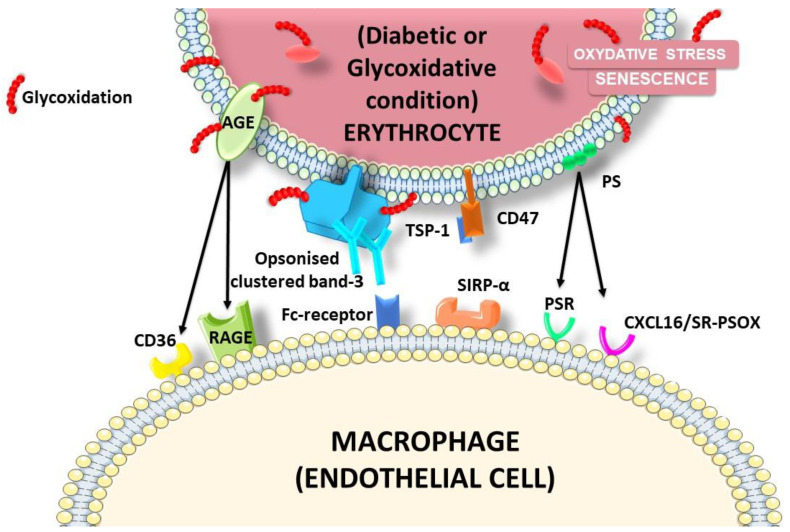
Ligands and receptors involved in glycated erythrocyte interactions with phagocytic cells. Several changes in the erythrocyte membrane are induced by glycation, such as PS exposure, CD47 shift, AGE formation and band.3 clustering. All these changes can be considered as ligands for the recognition and clearance of glycated/damaged erythrocytes.

Glycation-induced erythrocyte modifications initiate their adhesion to endothelial cells and their clearance by EP. Glycation causes an increase in eryptosis [171]. It has been shown that glycation and ageing promote erythrocyte adhesion and subsequent phagocytosis by endothelial cells [72]. This interaction and phagocytosis can occur via several receptors and ligands (Figure 11). Diabetes, characterised by high plasma glucose concentrations, and other diseases, such as malaria and sickle cell disease, are accompanied by membrane asymmetry and phosphatidylserine exposure in the outer leaflet of erythrocytes [206,207,208,209]. This phenomenon is known to serve as an “eat me” signal for the recognition and removal of the senescent cells by phagocytes [210]. Borst et al., demonstrated that this phosphatidylserine exposure by erythrocytes is also accompanied by an increase in their adhesion to the vascular endothelium in pathological conditions and appears to play a role in the development of cardiovascular complications by disturbing the circulation through narrow capillaries [211,212]. The clearance of erythrocytes bound to the vascular wall is thus a very important process. The binding of eryptotic and glycated erythrocytes to the vascular wall is in part due to phosphatidylserine exposure and their recognition by endothelial CxCL16, also called SR-PSOX, a surface-anchored chemokine described as a scavenger receptor known to bind oxidised LDL particles and phosphatidylserines [211,213,214].

AGEs also appear to mediate erythrocyte adhesion to the endothelium via their receptor AGE receptor (RAGE) or CD36. Indeed, under diabetic conditions or during ageing, glucose can react with erythrocyte proteins, leading to the reversible formation of AGEs with surface proteins. RAGE is expressed by a large number of cells, including macrophages and endothelial cells. In diabetes mellitus, band.3 is the most glycated protein, which binds to RAGE on endothelial cells [160]. The binding of AGEs to their receptors results in the promotion of oxidative stress, ROS production and inflammation. This could contribute to arterial ageing and LDL oxidation [215].

CD47 is a ubiquitously expressed cell surface glycoprotein acting as marker of self on erythrocytes. Erythrocytes devoid of CD47 are rapidly cleared from the bloodstream [216,217]. It functions as a “do not eat me” signal through its interaction with its receptor, SIRPα, on phagocytes. This interaction is known to inhibit EP. According to the findings of several studies, the ageing and/or oxidation of erythrocytes may induce a conformational change in CD47 and its subsequent binding to thrombospondin-1 (TSP-1). The recognition of the CD47-TSP1 complex by SIRPα will provide an eat me signal [218]. This suggests that CD47 can regulate the clearance of erythrocytes by macrophages by acting as a molecular switch for their phagocytosis and thus determine their life span.

The clustering of band.3 (also called anion exchanger 1) triggered by oxidative stress also plays an essential role in erythrocyte clearance. Indeed, upon oxidation and ageing, band.3 loses its anchorage to the cytoskeleton and forms clusters [219,220]. One of the pathways proposed is the formation of hemichromes (denatured haemoglobin) mediated by oxidative stress that will bind to band.3 cytoplasmic domains [219,221,222]. This binding of hemichrome, a source of oxidative stress, causes cross linking through SH bonds and the phosphorylation of tyrosine [219,220,223,224]. Band.3 will then be dissociated from other cytoskeletal proteins and form clusters [225]. This clustering phenomenon generates a high affinity site for the binding of autologous anti-band.3 antibodies. This opsonization enhances the phagocytosis of RBCs by macrophages. Indeed, autologous antibodies opsonizing band.3 are recognised by macrophages (FcR), leading to EP [223,226]. In diabetes mellitus, the band.3 protein can also be glycated and acquires the ability to bind to the RAGE [227].

To summarize, the glycation of erythrocytes in a chronic hyperglycaemic situation contributes to an increased rigidity of their membrane, thus facilitating their aggregability and altering their rheological properties. The reduced deformability of erythrocytes is associated with accelerated senescence and susceptibility to haemolysis. Whether intravascular or extravascular, this haemolysis exacerbated by the glycation phenomena will participate in the expansion of the plaque and eventually its rupture.

In the case of plaque rupture or vascular lesions, collagen and other extracellular matrix proteins are exposed, initiating platelet adhesion and thrombus formation [228]. Erythrocytes also participate in thrombus formation. Indeed, exposing erythrocytes to phosphatidylserine may activate coagulation enzymes and the assembly of coagulant complexes [229]. It has also been shown that senescent erythrocytes can bind platelets under flow conditions [182]. This binding occurs via phosphatidylserine at the erythrocyte membrane and CD36/CXCL16 at the surface of platelets [182]. Interactions between platelets and erythrocytes initiate thrombus formation and clotting. Indeed, the treatment of erythrocytes with lysophosphatidic acid, a phospholipid released from platelets during coagulation, induces the exposure of PS by erythrocytes and the release of procoagulant microvesicles [230,231].

## 5. Concluding Remarks

Intratissue haemolysis (erythrocyte lysis and EP) and its subsequent impact remain unclear and often mistakenly ascribed only as a consequence rather than a cause in the progression of a disease. However, some evidence has emerged about the importance of this process in pathological conditions, such as haemorrhagic stroke, cancer, tumour progression and atherogenesis.

Indeed, erythrocytes should certainly not be considered as simple bystanders in ATH, as they may constitute important actors at all steps of disease progression.

First, circulating erythrocytes exert a detrimental impact on the vascular endothelium, participating in the development of atheromatous plaques. Second, erythrocytes trapped within the plaque fuel the damaged vessel in lipids and pro-oxidant molecules, such as haemoglobin, heme and iron. Third, intraplaque macrophage iron handling due to the erythrocyte lysis and phagocytosis strongly contribute to the formation of foam cells and the destabilization of plaques. Finally, the resulting erythrocyte-mediated weakening of a plaque can lead to its rupture. In diabetic conditions, the proatherogenic potential of erythrocytes may be reinforced after modification by glycoxidation. Specific tools are mandatory to decipher the EP phenomenon of glycoxidised erythrocytes in vascular cells contributing to ATH. If glycoxidised erythrocytes may participate in ATH, their roles in other diabetic complications warrant particular attention. In stroke, are glycoxidised erythrocytes associated with adverse outcomes in diabetic patients? During retinopathology, do glycated erythrocytes participate in microvascular disease progression?

Further studies are needed to precisely document the occurrence and participation of glycoxidised erythrocytes in the development of vascular pathologies.

## Abbreviations

ATH	atherosclerosis
RBCs	red blood cells
EP	erythrophagocytosis
LDL	low-density lipoprotein
oxLDL	oxidised low-density lipoprotein
VSMC	Vascular smooth muscle cells
VCAM1	vascular cell adhesion molecule 1
ICAM1	Intercellular adhesion molecule1
Hb	haemoglobin
oxyHb	oxygenated form of human haemoglobin (or ferro Hb)
Hp	haptoglobin
Hx	hemopexin
HMOX1	heme oxygenase 1
Fpn	ferroportin
Hepc	hepcidin
PS	phosphatidylserine
DAMPs	Damage-Associated Signalling Molecule Patterns
NO	nitric oxide
eNOS	endothelial nitric oxide synthase
ROS	reactive oxygen species
O_2_	superoxide anion
H_2_O_2_	hydrogen peroxide
HO	hydroxyl radical
